# Phytogeographical and sociolinguistical patterns of the diversity, distribution, and uses of wild mushrooms in Côte d’Ivoire, West Africa

**DOI:** 10.1186/s13002-019-0284-5

**Published:** 2019-01-18

**Authors:** Bakary Soro, N’golo Abdoulaye Koné, Linda Patricia Louyounan Vanié-Léabo, Souleymane Konaté, Adama Bakayoko, Daouda Koné

**Affiliations:** 1 Université Nangui Abrogoua, UFR des Sciences de la Nature (UFR-SN), Unité de Recherche en Ecologie et Biodiversité (UREB), 28 BP 847 28, Abidjan, Côte d’Ivoire; 2 Centre de Recherche en écologie (CRE), Station de Recherche en Ecologie du Parc National de la Comoé, Bouna, Côte d’Ivoire; 3 University Félix Houphouët-Boigny, WASCAL Graduate Study Program Climate Change and Biodiversity, Centre d’Excellence Africain en Changement Climatique, Biodiversité et Agriculture Durable (CEA-CCBAD), 22 BP 582 Abidjan 22, Abidjan, Côte d’Ivoire

**Keywords:** Wild useful mushrooms, Diversity, Distribution, Sociolinguistic Indigenous knowledge, Ethnomycology, Côte d’Ivoire

## Abstract

**Background:**

Many fungal species in tropical Africa are useful, with high added value, and play essential roles in the structure and dynamic of ecosystems. However, the diversity, distribution, and uses by local populations of these non-timber forest products (NTFPs) and their respective habitats are still very poorly understood in sub-Saharan Africa in general and more specifically in Côte d’Ivoire. This study aims at (i) inventorying the wild useful mushrooms of Côte d’Ivoire within its major protected areas and their respective surrounding sociolinguistical groups, according to climatic and phytogeographical gradients, and (ii) recording ethnomycological knowledge and considerations of these local people.

**Methods:**

Field and ethnomycological surveys were conducted in the main and highest protected areas of Côte d’Ivoire (Comoé, Marahoué, and Taï national parks) and a set of their respective surrounding villages, along climatic and phytogeographical gradients. Standardized methods (permanent plots and opportunistic searches) were used for field surveys. In addition, a total 748 respondents belonging to 13 ethnic groups were interviewed at a rate of 300 interviewees during the preliminary investigations and 448 persons during the proper ethnomycological surveys.

**Results:**

Sixty-eight useful wild fungal species, belonging to 17 families and 23 genera, were listed and collected. Four categories of usage were reported by the rural people (food, medicinal, belief and recreational), with a dominance of food and medicinal uses. Fifty-six species were reported to be used as food and 16 species as medicinal fungi. These uses varied not only from one sociolinguistical group to another but also from a visited village to another. The high number (41) of the reported useful species was found in the Sudano-Guinean savanna zone while 28 species were collected in the forest zone and 22 species in the forest-savanna mosaic zone. These mushrooms were either saprotrophic or symbiotic (ectomycorrhizal or termitophilic). *Auricularia* sp3, *Psathyrella tuberculata*, and *Termitomyces* spp. were found as the most commonly used mushrooms.

**Conclusions:**

These national scale field and ethnomycological surveys give one of the more complete but non-exhaustive list of useful mushrooms of Côte d’Ivoire. Mushrooms are relatively well known and used by the Ivorian people within the main phytogeographical zone of the country. These people also have an interest in all the functional groups with an important phytogeographical zone-fungal-specific used species. However, protected areas of the visited zones seem to represent the last sanctuaries of these organisms due to high rate of loss of natural habitats.

**Electronic supplementary material:**

The online version of this article (10.1186/s13002-019-0284-5) contains supplementary material, which is available to authorized users.

## Background

The diet of West African people partly depends on edible products from animals (caterpillar, snail), plants (leaves, fruits), or mushrooms [1]. There is a renewed of interest for such products mainly due to the high cost of living, imported (wheat flour, rice) and local agriculture’s products (beans, corn, millet). Wild edible mushrooms are harvested in huge quantities, widely consumed and sold during their respective fructification periods, in Eastern [2, 3], Central [4, 5], and Western [6, 7] Africa. These organisms represent a real additional source of income for rural women and young [7]. In addition to the fact of being appreciated for their nutritional values [8, 9], these non-timber forest products (NTFPs) are at the origin of several dietary, ethnic, cultural, religious, or medicinal considerations depending on the ethnic groups [10, 11].

The global diversity of fungi is estimated to be 1.5 million species worldwide [12, 13]. However, only 7% of this diversity is known [14]. The percentage of this known diversity hardly exceeds 3% in West Africa [15]. However, within Africa, edible mushrooms are relatively well known in Central, Southern, and Eastern Africa [3, 16–18]. In sub-Saharan Africa, roughly 300 edible mushrooms have been identified and described even tough an annual consumption of more than 30 kg per rural inhabitant, particularly in Central and Southern Africa [6, 19, 20]. Furthermore, on a total of 151 references on African edible mushrooms, only three are related to West Africa [21–23].

The practical and applied interests of mushrooms are huge [14], in human nutrition (wild and cultivated edible species), agriculture, and forestry (bioindicator species, regulation of ecosystem functioning and dynamic, or uses in forest regeneration), industry (species used in biotechnology), pharmacy and medicine (species used in medicine), etc. However, some fungal species (i.e., *Termitomyces* spp.) are declining in abundance and diversity from year to year, due (1) to their bad and uncontrolled harvest for consumption (trade) and secondly (2) to the increasing reduction of their living habitats mainly attributable to forest fragmentation, shifting slash and burn cultivation, and extensive farming [7]. Furthermore, selective (focused on specific tree species) and/or massive cutting down of woodlands is not only inevitably accompanied by qualitative erosion of wild mushroom diversity but also by the drastic reduction in the quantitative availability of fungal food resources (cf. edible ectomycorrhizal mushrooms). For example, 28 endangered edible species were recorded in Benin, due to the permanent reduction of their habitats [24]. Côte d’Ivoire for its part is characterized by an annual loss of natural ecosystems of around 300,000 ha/year [25]. This massive destruction of habitats inevitably leads to serious losses of useful plants, mushrooms, and animal resources. This is a real threat to the survival of local people who exploit such resources on a daily basis. However, various phytogeographical zones are encountered in Côte d’Ivoire; including the Guinean, Sudano-Guinean, and Sudanese zones. This phytogeographical heterogeneity includes diverse vegetation types which in turn strongly influence mycoflora [6, 26]. There is no doubt that such mycofloristic variability and its potential consequent indigenous ethnomycological knowledge exist in Côte d’Ivoire.

This study aimed at (i) inventorying the wild useful mushrooms of Côte d’Ivoire within its major protected areas and their respective surrounding sociolinguistical groups according to a climatic and phytogeographical gradients and (ii) recording ethnomycological knowledge and considerations of these local people. In sum, the hypothesis that the diversity of mushrooms in Côte d’Ivoire, the ethnomycological knowledge of the Ivorian people, and their uses of these mushrooms are phytogeographical and sociolinguistical group dependent.

## Methods

### Study areas: the Ivorian national parks and some of their respective surrounding villages

**Fig. 1:**
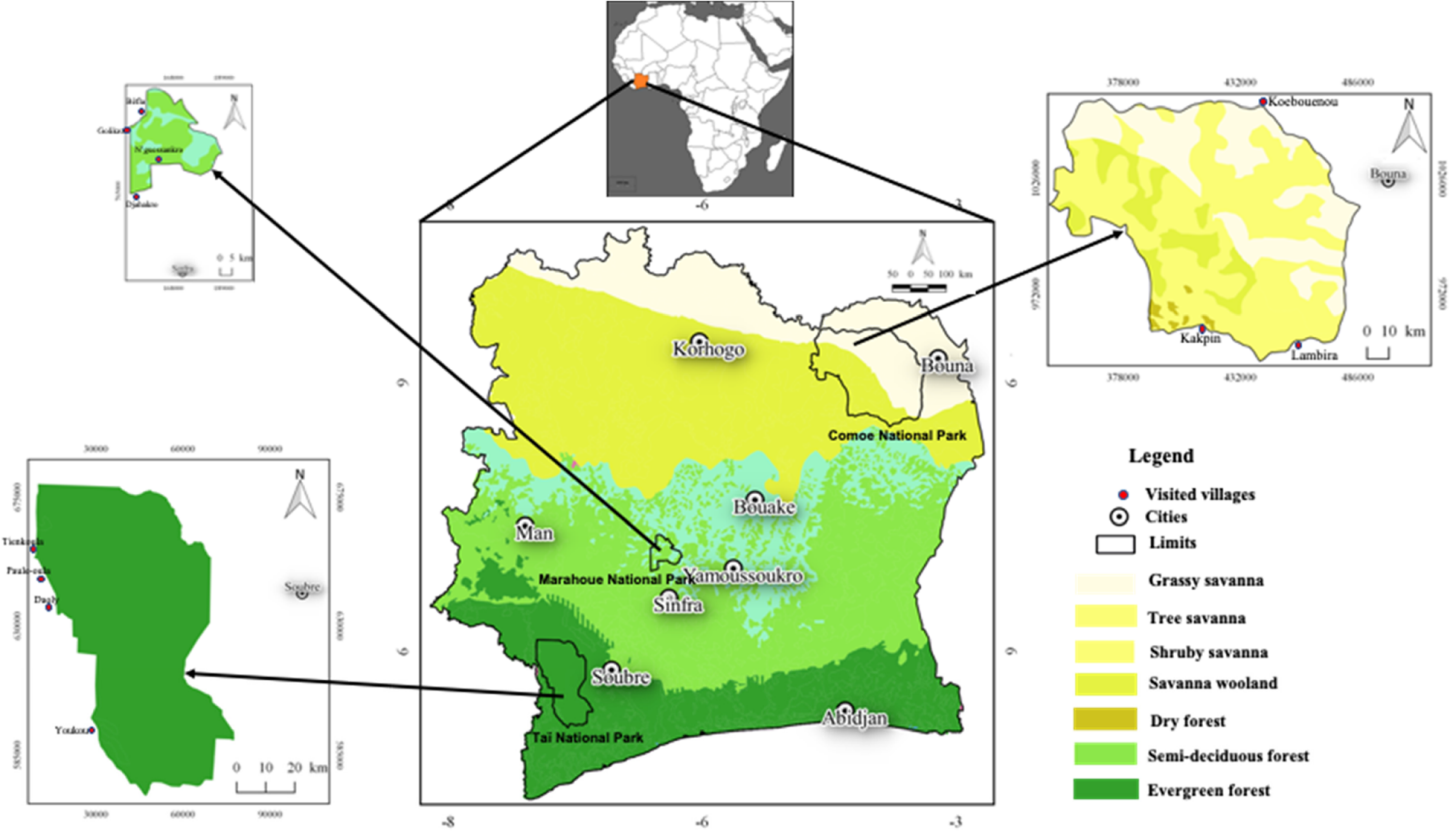
Location of the study areas

This work was done in three national parks (Taï, Marahoué, and Comoé) and their surrounding areas, according to a phytogeographical and climatic gradient in Côte d’Ivoire (West Africa). The Fig. 1 presents the location of these study areas while the Table 1 shows the characteristics of each visited park with its respective vegetation types, visited villages, and ethnic groups interviewed during ethnomycological surveys.

**Table 1:** Characteristics of the study areas

Study zones	Characteristics	Visited villages	Interviewed sociolinguistical groups
Evergreen Forest zone (EF)/Taï National Park (TNP)	Status: UNESCO World Heritage Site and Biosphere Reserve Location: Southwestern Côte d’Ivoire (5° 08′–6° 24′ N and 6° 47′–7° 25′ W) Size: 4540 km^2^ Vegetation: upper Guinean evergreen forest. Climate: annual rainfall ranges from a mean of 1700 mm in the north to 2200 mm in the southwest, falling from March/April to July, with a shorter wet season from September to October. Particularity: the largest island of forest remaining in West Africa, remaining relatively intact and well conserved. Main anthropogenic impacts: threatened mammal species such as the pygmy hippopotamus and 11 species of monkeys are of great scientific interest. New main region of cacao production of the country	Paulé-oula, Youkou, Tienkoula, Daobly	Oubi, Baoulé, Gouro, Sénoufo, Kroumen, Guéré
Guinean Savanna Zone (GS)/Marahoué National Park (MNP)	Status: Endangered National Park Location: West-center (7° 05′ 49′ N–6° 01′ 32′ W) Size: 1010 km^2^ Vegetation: forest-savanna mosaic (Guinean savanna) Climate: mean annual precipitation varies between 1100 and 1800 mm. Two dry periods can be distinguished, stretching from November to February and from July to August. Particularity: covered with forest (2/3) and savanna. Four main habitats types occur: open canopy forest, gallery forests, forest-savanna edge, and savannas (savanna woodland, tree savannas, and shrubby savannas). Main anthropogenic impacts: illegal farming (illegal farms cover huge areas in the former forest zone), poaching, and fire. It has lost 93% of its forest cover in the last 6 years.	Bêfla, Golikro, Djahakro, N’guessankro	Gouro, Baoulé, Whan
Sudano-Guinean savanna zone (SGS)/Comoé National Park (CNP)	Status: UNESCO World Heritage site and Biosphere Reserve Location: North-eastern (8° 30′–9° 40′ N and 3° 10′–4° 20′ W). Size: 11,500 km^2^ Climate: mean annual precipitation is around 1150 mm with a mean annual temperature of 27 °C. Vegetation: semi-natural mosaic of forest-savanna (Sudano-Guinean savanna). Particularity: many habitats ranging from forests to savannas, including all types of savanna (84%), bowal (4.9%), gallery forest (2.3%), and dry and humid forest islands Main anthropogenic impacts: uncontrolled annual fire	Kakpin, Lambira, Koebounou	Koulango, Lobi, Malinké, Lorhon

### Choosing study sites and identifying mycophagous ethnic groups

Preliminary ethnomycological investigations were carried out on 300 people, at the rate of 100 persons per phytogeographical zone, for the determination of the indigenous knowledge of respective communities. The questionnaires and casual conservations were structured around five main points: (i) the availability of wild mushrooms in the visited localities; (ii) indigenous knowledge and especially the uses of wild mushrooms by local populations; (iii) diversity and abundance of wild useful mushrooms; (iv) the market value of these wild useful mushrooms and the identification of actors of a potential seasonal trade of these fruit bodies; (v) fructification periods and habitats of these wild edible and medicinal mushrooms. This work has been done through household surveys and semi-structured interviews. These preliminary surveys were followed by a first field sampling surveys within the visited areas.

After the preliminary investigations and the first field sampling surveys, a common photo album was prepared with photos of all identified wild mushroom species and their respective habitat. This photo album was used for the proper ethnomycological surveys on 448 people [27], within the identified mycophagous ethnic groups of each phytogeographical zone. These surveys were conducted in the households, late in the afternoon or in the evening at farmers’ home. Moreover, the rural markets, roads, and tracks leading to farms and localities bordering each national park were visited. These surveys were carried out by local assistants previously trained under our supervision during the preliminary investigations. These local assistants were selected in the villages on the basis of their botanical, mycological (vernacular names of trees and mushrooms ), and especially local language knowledge.

These proper ethnomycological surveys were mainly based on semi-structured interviews and casual conversations with rural people in the visited villages. The semi-structured interviews were based on previously defined questions while casual conversations were occasional. The questionnaires and casual conversations were structured around (i) the availability and diversity of the wild useful mushrooms, (ii) the specific uses of each reported species, (iii) the fructification periods and habitats of each reported species, and (iv) the fungal harvesting methods.

The choice of the villages to be visited was based on the cultural considerations of the local people; not on their arrangement in the vicinity of the visited protected areas.

### Sampling design and visited habitat types

Rapid assessment protocol via opportunistic searches was used for fungi sampling. Fruit bodies of wild mushrooms were collected in the national parks and their respective natural and anthropized surrounding habitats from 2014 to 2017. These opportunistic searches were completed with specific sampling methods respectively implemented within each phytogeographical zone:

•In the CNP, a total of 12 permanent plots of 30 m × 30 m were established within 4 habitat types at a rate of 3 plots per habitat type. A gallery forest and tree habitats dominated by plants of Caesalpiniaceae and Phyllantaceae families were visited. Nine permanent plots were established in *Isoberlinia doka* Craib and Stapf, *Uapaca togoensis* Baill., and mixed woodlands (*Isoberlinia doka* Craib and Stapf + *Uapaca togoensis* Baill.) at a rate of tree plots per habitat type. Tree additional plots were established in a gallery forest;

•In the MNP, only opportunistic searches were undertaken within forests (forest islands, gallery forest) and savannah woodlands;

•In the TNP, nine plots of 30 m × 30 m were established in strictly protected and rural evergreen forest habitats. Some opportunistic searches and collections were also done within some anthropized habitats such as cocoa, coffee, food crop plantations, and old fallows.

Photographs of the specimens were made in situ and macroscopic features’ descriptions of the collected fruit bodies made on fresh samples using the standardized description form by [4] in order to record evanescent or changing features when drying. The specimens were then dried at a temperature of 40 °C for 24 to 48 h and then packaged in ‘Minigrip’ plastic bags for future microscopic and anatomic studies.

### Indigenous knowledge and identification of useful wild mushrooms

Ethnomycological knowledge within sociolinguistical groups was assessed by calculating the use frequencies of each reported fungal species within each use category. These values allowed determining scores for the calculation of some ethnomycological indices based on the 448 informants of the proper ethnomycological surveys:

•The reported use value (RUV) at the sociolinguistical group level;

•The ethnomycological use value of a species for a category of use (EUVk);

•The Cultural Significance Index (CSI) helped assessing and comparing the indigenous knowledge of useful fungal species within and between the visited sociolinguistical groups for the identification of the most used species per village and sociolinguistical group [28–30].

The first identifications and indigenous knowledge on the edibility and uses of the collected samples were made by the trained local assistants directly in the field. These fresh pre-identified specimens were then showed to the local populations for further indigenous knowledge (vernacular names, uses, and habitats). Furthermore, the photo album designed at the end of preliminary ethnomycological investigations and the first field works was also used. Every new indigenous knowledge or information (uses, vernacular name and its significance, and habitats) was judged as reliable after obtaining it from at least 75% of the informants within a specific sociolinguistical group and visited village.

### Identification of specimens

The macroscopic and microscopic descriptions were made from exsiccata (dried fruit bodies) using conventional taxonomy techniques in mycology with a standardized description grid. These identifications were made on the basis of macro and microscopic observations, by consulting mushroom herbaria, field mushroom guides, and other standard-related available literature for *Termitomyces* species [3, 7, 31–33] and others species [4, 19, 34, 35].

## Results

A total of 448 informants were interviewed during the proper ethnomycological survey, namely, 145 informants (32.37%) in the Evergreen Forest zone, 131 informants (29.24%) in the Guinean savanna zone, and 172 informants (38.39%) in the Sudano-Guinean savanna zone. The informants were mainly women, not only at the total interviewees level (63,62%) but also at the phytogeographical zone level (i.e., 20.98% in the EF, 15.85% in GS, and 26.79% in SGS). Furthermore, informants of each ethnic group represent at least 12% of the total interviewees. The age of the informant range from 12 to 70 years.

### Diversity, abundance, and distribution of wild edible and medicinal mushrooms

Sixty-eight wild edible and medicinal mushrooms species belonging to 23 genera and 17 families were recorded and identified (Table 2). These species belong to the functional groups of saprotrophic (38.70%) and symbiotic (60.30%) fungi. The symbiotic group was constituted of termitophilic (22.05%) and ectomycorrhizal (38.23%) fungal species. The diversity of these useful fungal species was found different from a phytogeographical zone to another.

**Table 2:** Diversity and uses of useful wild mushrooms of Côte d’Ivoire

Species	Families	Voucher number	Distribution	Reported uses	RUV	CII
*Agaricus* sp1	Agaricaceae	SB190	EF	Food	0.41	0.29
*Agaricus* sp2		SB206	EF	Food	0.36	0.22
*Amanita congolensis* (Beeli) Tulloss, B. E. Wolfe, K. W. Hughes, Kudzma and Arora	Amanitaceae	LLPV110	SGS	Food	0.57	0.77
*Amanita masasiensis* Härk. and Saarim.		LLPV620	SGS	Food	0.50	0.38
*Amanita strobilaceovolvata* Belli		LLPV233	SGS	Food	0.71	0.71
*Amanita aff subviscosa* Belli		LLPV218	SGS	Food	0.55	0.50
*Amanita craseoderma* Bas		LLPV112	SGS	Food	0.25	0.27
*Amanita crassiconus* Bas		LLPV333	SGS	Food	0.27	0.21
*Amanita rubescens* Pers*.*		LLPV132	SGS	Food	0.52	0.43
*Amanita xanthogala* Bas		LLPV159	SGS	Food	0.36	0.37
*Auricalaria polytricha* (Bull.) Quél.	Auriculariaceae	SB186	EF, GS, SGS	Food and medicinal	0.32	0.25
*Auricularia* sp1		SB101	EF, GS, SGS	Food and medicinal	0.57	0.48
*Auricularia* sp2		SB104	EF, GS, SGS	Food and medicinal	0.61	0.61
*Auricularia* sp3		SB051	EF, GS, SGS	Food and medicinal	0.51	0.42
*Boletus loosii* Heinem.	Boletaceae	LLPV1002	SGS	Food	0.34	0.21
*Bulgaria* sp.	Bulgariacea	SB184	EF, GS	Medicinal	0.29	0.37
*Cantharellus addaiensis* Henn.	Cantharellaceae	LLPV100	SGS	Food	0.87	0.87
Cookeina sp1	Sarcosyphaceae	SB156	EF	Medicinal	0.02-	0.03
Cookeina sp2		SB193	EF	Medicinal	0.05	0.05
Coprinus africanus (Pegler) Redhead, Vilgalys and Moncalvo	Agaricaceae	SB 217	EF, GS, SGS	Food	0.66	0.59
*Daldinia concentrica* (Bolton) Cesati and de Notaris	xylariaceae	SB189	EF, GS, SGS	Medicinal and magic	0.64	1.11
*Echinochaete brachypora* (Mont.) Ryvarden	Polyporaceae	SB073	EF, SGS	Food and medicinal	0.28	0.15
*Ganoderma lucidum* (Curtis ex Fr.) P. Karst.	Ganodermataceae	SB007	SGS	Medicinal	0.61	0.42
*Ganoderma* sp.		SB210	EF	Medicinal	0.07	0.02
*Gyroporus castaneus* (Bull.) Quél.	Gyroporaceae	LLPV442	SGS	Food	0.43	0.42
*Lactarius saponaceus* Verbeken	Russulaceae	LLPV079	SGS	Food	0.23	0.24
*Lactarius tenellus* Verbeken and Walleyn		LLPV902	SGS	Food	0.22	0.34
*Lactifluus flammans* (Verbeken) Verbeken		LLPV188	SGS	Food	0.72	0.67
*Lactifluus gymnocarpoides* (Verbeken) Verbeken		LLPV199	SGS	Food	0.19	0.18
*Lactifluus heimii* (Verbeken) Verbeken		LLPV164	SGS	Food	0.61	0.78
*Lactifluus luteopus* Verbeken		LLPV191	SGS	Food	0.70	0.58
*Lactifluus volemoides* (Karhula) Verbeken		LLPV189	SGS	Food	0.67	0.67
*Lentinus squarrossulus* Mont.	Polyporaceae	SB001	EF, GS, SGS	Food	0.94	0.28
*Lentinus tuber-regium* (Fr.) Singer		SB093	EF, GS	Food and medicinal	0.2	0.28
*Lycoperdon* sp1	Agaricaceae	SB188	EF	Food and medicinal	0.11	0.07
*Lycoperdon* sp2		SB138	EF	Food and medicinal	0.12	0.06
*Marrasmiellus inoderma* (Berk.) Singer	Marasmiaceae	SB098	EF, GS	Food	0.54	0.20
*Marasmiellus* sp.		SB042	EF, GS	Food	0.21	0.12
*Octaviana ivoryana* Casttelano, Verbeken and Thoen	Boletaceae	LLPV214	SGS	Positioning	0.15	0.20
*Psathyrella tuberculata* (Path.) A. H. Smith	Psathyrellaceae	SB026	EF, GS, SGS	Food and medicinal	1.20	1.81
*Psathyrella* sp.		SB205	EF, GS, SGS	Food	0.63	0.58
*Russula aff cellulata* Buyck	Russulaceae	LLPV118	SGS	Food	0.49	0.48
*Russula ciliata* Buyck		LLPV123	SGS	Food	0.47	0.49
*Russula congoana* Pat.		LLPV048	SGS	Food	0.37	0.43
*Russula grisea* Fr*.*		LLPV553	SGS	Food	0.51	0.53
*Russula oleifera* Buyck		LLPV187	SGS	Food	0.51	0.47
*Russula sesenagula* Beeli		LLPV127	SGS	Food	0.32	0.35
*Russula* sp.		SB41	GS	Food	0.36	0.40
*Schizophyllum commune* Fries	Schizophyllaceae	SB061	EF, GS	Food	0.07	0.07
*Termitomyces* cf *Aurantiacus* (R. Heim) R. Heim	Lyophyllaceae	NAK170	SGS	Food	0.65	0.73
*Termitomyces* cf *striatus* (Beeli) R. Heim		SB043	SGS	Food	0.82	1.10
*Termitomyces fuliginosus* Heim		NAK004	SGS	Food	0.85	0.87
*Termitomyces letestui* (Pat.) R. Heim		NAK001, SB30	GS	Food	0.69	1.45
*Termitomyces meduis* R. Heim and Grassé		NAK003, SB086	EF, GS, SGS	Food	0.77	0.98
*Termitomyces eurhizus* (Berk.) R. Heim		NAK002	EF	Food	0.25	0.36
*Termitomyces microcarpus* (Berk. and Broomo) R. Heim		NAK079	EF	Food	0.64	0.87
*Termitomyces schimperi* (Pat.) R. Heim		NAK103	EF	Food	0.69	0.62
*Termitomyces* cf *clypeatus*		NAK019	EF	Food	0.61	0.72
*Termitomyces* sp1		SB095	GS	Food	0.87	1.19
*Termitomyces* sp2		SB043	GS	Food	0.64	0.91
*Termitomyces* sp3		SB004	GS	Food	0.82	1.11
*Termitomyces* sp4		SB012	GS	Food	0.85	1.12
*Termitomyces* sp5		NAK037	GS	Food	0.70	1,07
*Termitomyces* sp6		SB10	GS	Food	0.86	1.15
*Termitomyces* sp7		NAK097	GS	Food	0.41	0.62
*Volvariella earlei* (Murrill) Shaffer	Volvariellaceae	SB013	EF, GS, SGS	Food	0.60	0.64
*Volvariella volvacea* (Bull.) Singer		SB 216	EF, GS, SGS	Food and medicinal	0.85	1.43
*Volvariella* sp.		SB041	GS	Food	0.06	0.08

*Abbreviations: zones* phytogeographical zones of Côte d’Ivoire, *EF* Evergreen Forest, *GS* Guinean savanna, *SGS* Sudano-Guinean Savanna, *RUV* reported use value, *CSI* Cultural Significance Index

**Fig. 2:**
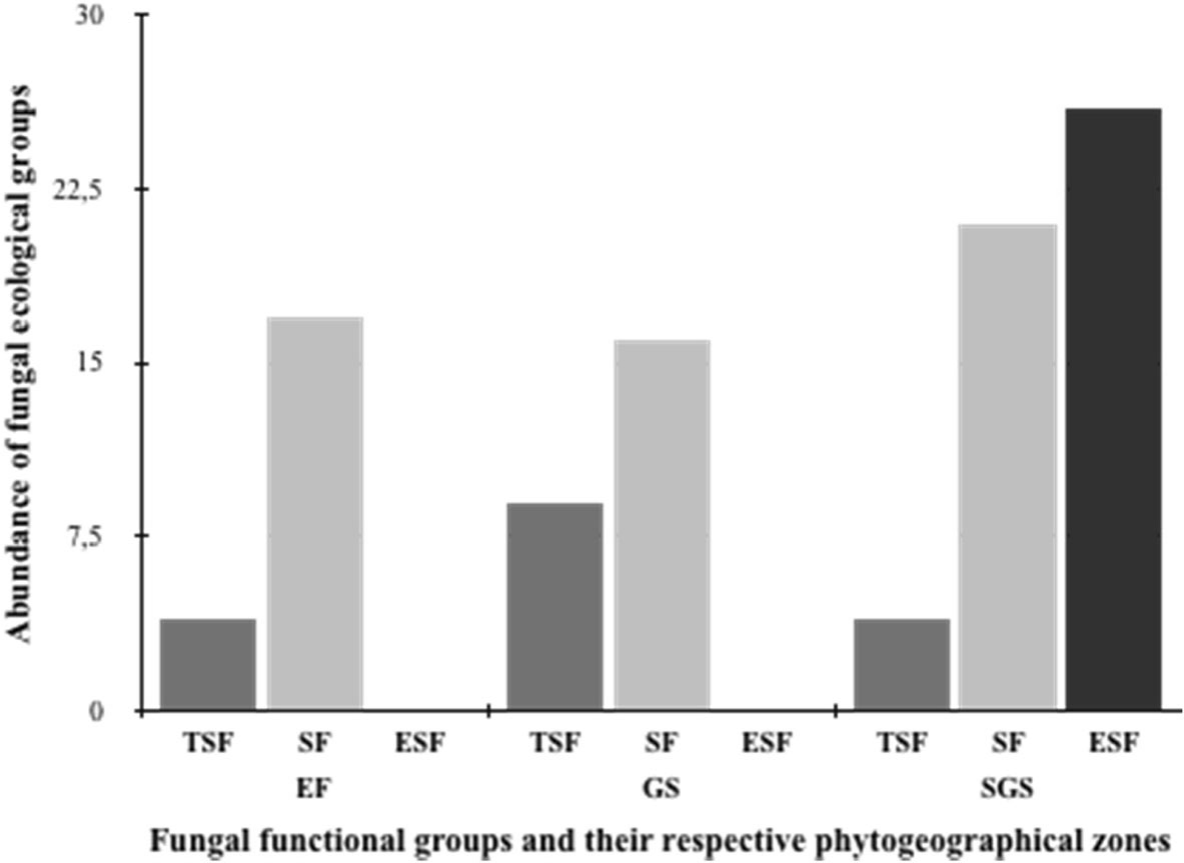
Abundance of the recorded wild useful fungi according to their respective ecological group and phytogeographical zone. Abbreviations: TSF = termitophilic symbiotic fungi, ESF = ectomycorrhizal symbiotic fungi, SF = saprophytic fungi; EF = Evergreen Forest, GS = Guinean Savanna, SGS = Sudano-Guinean savanna

Forty-one species were recorded in the Sudano-Guinean savanna zone while 28 species were identified in the Evergreen Forest zone and 22 species the Guinean savanna zone. The Sudano-Guinean savanna zone was dominated by the ectomychorizzal (Russulaceae and Amanitaceae) and termitophilic (Lyophyllaceae: *Termitomyces* spp.) species; while the extend forest-savanna boundaries (Guinean savanna) and the forest zone were dominated by the termitophilic and saprotrophic fungal species (Fig. 2). Most of the saprotrophic fungi were common to the visited phytogeographical zones as well as a termitophilic symbiotic species (*Termitomyces medius*) (Table 2).

The Cultural Significance Index values (CSI) showed a significant difference of the reported wild useful mushrooms’ diversity between the interviewed sociolinguistical groups on the one hand and the visited villages on the other hand. Within the Evergreen Forest zone, in the localities of Paulé-oula and Daobly, the most used species belong is *Auricularia* sp3 by the Oubi and Gueré while *Psathyrella tuberculatta* and *Volvariella volvacea* species were identified as the most consumed species by the Baoulé. In the Guinean savanna zone, *Psathyrella tuberculata* was the most used mushroom by the Baoulé and Gouro in the localities of Djahakro and Golikro. This species was also identified as the most used fungal species by the Koulango, Malinké, and Lobi in the localities of Kapkin and Lambira within the Sudano-Guinean savanna zone.

### Reported uses of wild mushrooms in Côte d’Ivoire

Four categories: food, medicinal, belief (positioning) and myth (religious), and recreational (decoration and game) of usage were reported by the rural people with a dominance of food and medicinal uses. Whatever the phytogeographical zone, mushrooms were more used in diet than in local medicine. Fifty-six species were identified as food, and 16 species were used in local medicine (Table 2). *Lycoperdon* sp1 and *Lycoperdon* sp2 are used as games by kids while *Ganoderma* sp. is used for decoration, and *Octaviana ivoryana* Casttelano, Verbeken, and Thoen is used for positioning by hunters and poachers.

**Fig. 3:**
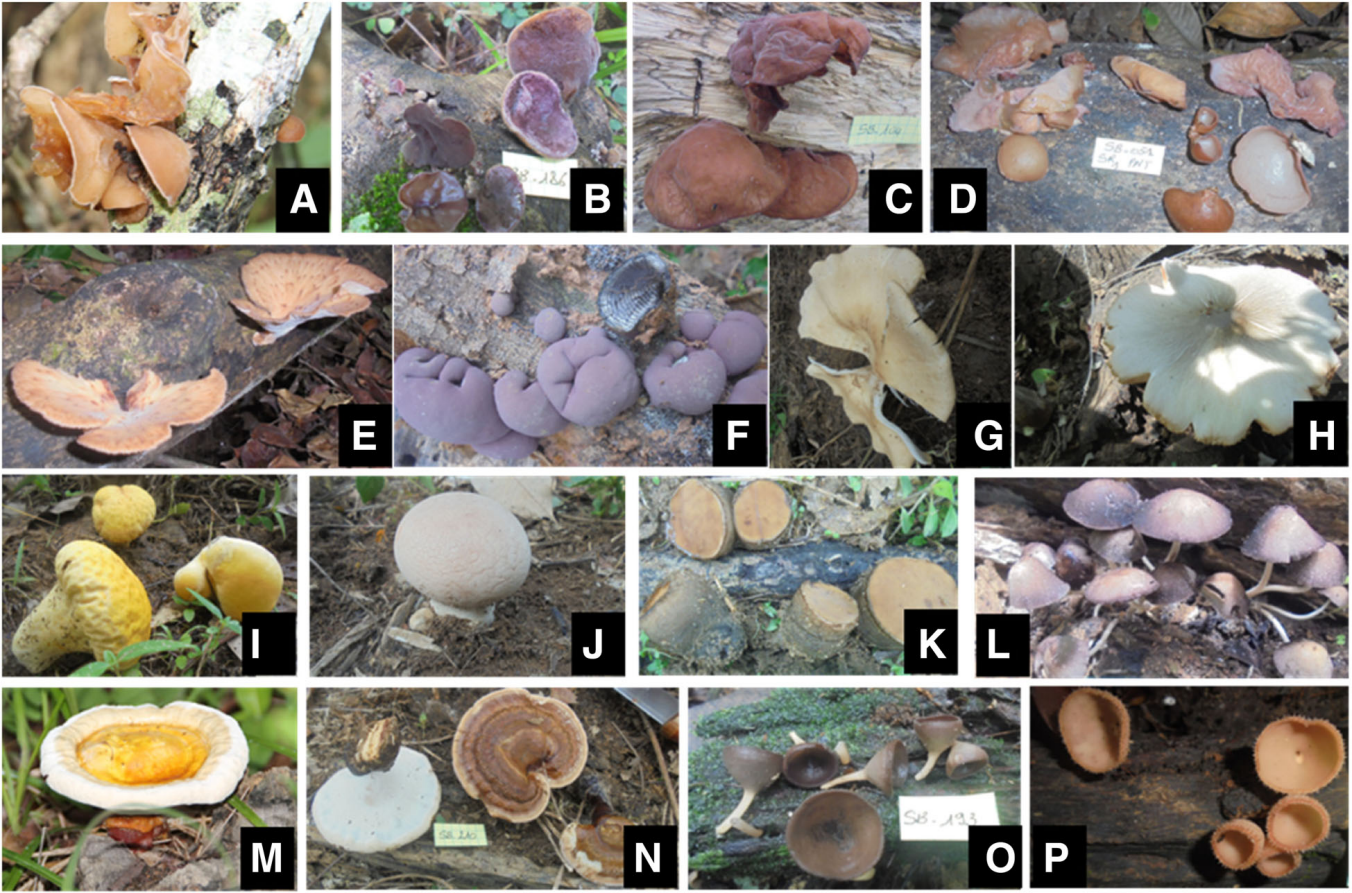
Reported and recorded wild medicinal fungi of Côte d’Ivoire. **a** *Auricularia polytricha* (Mont.) Sacc. **b** *Auricularia* sp1. **c** *Auricularia* sp2. **d** *Auricularia* sp3. **e** *Echinochaete brachypora* (Mont.) Ryvarden. **f** *Daldinia concentrica* (Bolton) Cesati and de Notaris. **g** *Lentinus tuber-regium* (Fries) Fries. **h** *Volvariella volvaceae* (Bulliard) Singer. **i** *Lycoperdon* sp1. **j** *Lycoperdon* sp2. **k** *Bulgaria* sp. **l** *Psathyrella tuberculata* (Path.) A. H. Sm. **m** *Ganoderma lucidum* (Curtis ex Fr.) P. Karst. **n** *Ganoderma* sp. **o** *Cookeina* sp1, **p** *Cookeina* sp2

A total of 16 wild medicinal species belonging to 9 families (Agaricaceae, Auriculariaceae, Ganodermataceae, Hypoxylaceae, Phacidiaceae, Plyteaceae, Polyporaceae, Psathyrellaceae, Sarcosyphaceae) were inventoried during this work (Fig. 3). *Auricularia polytricha* (Mont.) Sacc., *Auricularia* sp1, *Auricularia* sp2, *Auricularia* sp3, *Echinochaete brachypora* (Mont.) Ryvarden, *Daldinia concentrica* (Bolton) Cesati and de Notaris, *Lentinus tuber-regium* (Fries) Fries, *Volvariella volvacea* (Bulliard) Singer, *Lycoperdon* sp1, *Lycoperdon* sp2, *Bulgaria* sp., *Psathyrella tuberculata* (Path.) A. H. Sm., *Ganoderma lucidum* (Curtis ex Fr.) P. Karst., *Ganoderma* sp., *Cookeina* sp1, and *Cookeina* sp2 were reported as medicinal mushrooms. These mushrooms were reported to be used for the treatment of 28 illnesses (Table 3). However, most of the reported medicinal mushrooms were found usually used for the treatment of peptic ulcer (i.e., *Auricularia polytricha* (Mont.) Sacc., *Auricularia* sp1, *Auricularia* sp2, *Auricularia* sp3, *Bulgaria* sp., *Psathyrella tuberculata* (Path.) A. H. Sm.). Finally, interviewees were rather extremely discreet with the formula and the mode of administration of these reported medicines.

**Tables 3:** Diversity and distribution of the reported wild medicinal mushrooms according to the phytogeographical and sociolinguistical groups in Côte d’Ivoire

Fungal species	Families	Distribution	Sociolinguistical groups	Treated disease(s)
*Auricularia polytricha* (Mont.) Sacc.	Auriculariaceae	GS	Gouro	Peptic ulcer and hypertension
		EF	Guéré and Oubi	Menopause opportunistic diseases
*Auricalaria* sp1		SGS	Lobi and Malinké	Peptic ulcer
*Auricularia* sp2		SGS	Lobi and Malinké	Peptic ulcer
*Auricularia* sp3		EF	Guéré and Oubi	Menopause opportunistic diseases, peptic ulcer, HIV/AIDS
*Bulgaria* sp.	Phacidiaceae	EF	Baoulé	Heart condition
		EF, GS	Baoulé	Heart condition, peptic ulcer, epilepsy
*Cookeina* sp1	Sarcosyphaceae	EF	Guéré	Earache and hiccough
*Cookeina* sp2				
*Daldinia concentrica* (Bolton) Cesati and de Notaris	Hypoxylaceae	EF, GS, SGS	Baoulé, Gueré	Hernia, stomach ache, pediatrics (stomach ache), reduction of big navel of newborn babies, invigoration of kidney’s activity
		EF	Lobi, Oubi, Koulango, Gouro, Malinké, Guéré	Healing wound, boil, diarrhea, STD (gonorrhea), cheloids
*Ganoderma lucidum* (Curtis ex Fr.) P. Karst.	Ganodermataceae	SGS	Lobi, Koulango, and Malinké	Stomach ache, make giving birth easier, use as antibiotic after giving birth, hemorrhoid, healing wound, rheumatism, insecticide
*Ganoderma* sp.		EF	Guéré	Pediatrics (whet new born appetite)
*Lentinus tuber-regium* (Fries) Fries	Polyporaceae	GS	Baoulé	Pediatrics, burn’s wound
*Lycoperdon* sp1	Agaricaceae	EF	Guéré and Baoulé	Burn, pediatrics (whet new born appetite), reduction of big navel of newborn babies
*Lycoperdon* sp2				
*Psathyrella tuberculata* (Path.) A. H. Sm.	Psathyrellaceae	EF, GS, SGS	Baoulé, Oubi, Gouro, Koulango, Lobi, Malinké	Peptic ulcer
*Echinochaete brachypora* (Mont.) Ryvarden	Polyporaceae	EF	Baoulé	Heart condition
*Volvariella volvaceae* (Bulliard) Singer	Plyteaceae	GS	Baoulé	Fat removal

*Abbreviations*: *zones* phytogeographical zones of Côte d’Ivoire, *EF* Evergreen Forest, *GS* Guinean Savanna, *SGS* Sudano-Guinean Savanna, *STD* sexually transmitted diseases, *HIV/AIDS* human immunodeficiency virus/acquired immunodeficiency syndrome

### Vernacular nomenclature

Each of the identified useful fungal species has a specific vernacular name within every sociolinguistical group. Indeed, only useful species were found with vernacular names. These names were given according to the ecology (palm mushroom referring to *Volvariella volvacea*), the presence or absence of pseudorhiza (long stipe mushroom referring to all *Termitomyces* species), the resemblance to an item of everyday life, the color, the taste, the capacity, and specific use by a particular ethnic group or foreign person. In the Sudano-Guinean savanna zone, the vernacular names of ectomycorrhizal species were generally given according to the host plant species (Additional file 1: Table S1). However, some vernacular names reported by the interviewees had no meaning. Furthermore, misidentifications of some genera having common features (i.e., *Amanita and Volvariella*) lead to confusions in some sociolinguistical groups. This confusion was greater in the same genus, especially with species of the genus *Termitomyces*, in which all the species have the same vernacular name referring to their symbiosis with termites or to the presence of a pseudorhiza (long stipe).

## Discussion

Sixty-eight wild useful mushrooms were inventoried during this study. Previous studies [6] revealed 26 useful species in Côte d’Ivoire. This study gives a more complete and not exhaustive list of useful mushrooms of this country. This result might be due not only to the diversity of the visited phytogeographical zones and their respective ethnic groups but also to the long duration of data collection. Indeed, the specific diversity of wild edible mushrooms in countries is not only linked to a series of factors such as the size of the country, the diversity of ecosystems, the country’s floristic composition but also the diversity of ethnic groups [6]. Furthermore, this diversity is high than those of some central and west African countries. According to [36], 42 species were inventoried in Katanga in the Democratic Republic of Congo; while in West Africa, non-exhaustive lists indicated respective diversities of 56 species in Benin, 40 species in Burkina Faso, 45 species in Senegal, and 38 species in Togo [6, 11, 37–39]. Moreover, the diversity of these mushrooms was found differing from a phytogeographical zone to another, with the greatest observed diversity in the Sudano-Guinean savanna zone. Indeed, this phytogeographical zone is dominated by ectomycorrhizal plant species and also includes forest islands and gallery forests. This diversity of ecosystems favors the occurrence of a wide variety of mushrooms especially ectomycorrhizal and *Termitomyces* species.

Ectomycorrhizal mushrooms with very specific fructification habitats [6] were recorded only in the Sudano-Guinean savanna zone and where found known only by the populations living in the surrounding areas of the visited park (i.e., Comoé National Park). In contrast, saprotrophic and symbiotic mushrooms were found common to all the visited phytogeographical zones and represent the most used species by rural populations. Fruit bodies of these fungi occur in wooded habitats (i.e., forest for saprotrophic mushrooms and forest-savanna mosaic phytogeographical zone for *Termitomyces* species). This clearly shows that the identification and use of wild mushrooms depend on the phytogeographical zone but especially on the diversity of the mycophagous people found in every zone and the respective state of awareness. For example, the genus *Auricularia* is widely used by the Ouby and Guéré while it is rejected by the Baoulé in the same phytogeographical zone in Côte d’Ivoire. Species of this genus are also highly appreciated by the Bofi Pygmies of the Central African Republic [5] and the Bariba in Northern Benin [39].

Four categories: food, medicinal, belief and myth (religious, anemometer), and recreational (ornamentation and game) of usage were reported by the rural people with a dominance of food and medicinal uses. However, the majority of inventoried species are used as food during the whole year (i.e., intense periods of farm works and the dry seasons). Sixteen wild medicinal species were reported by the interviewees. These mushrooms were considered by local populations as contributing to their physical and psychological well-being. However, this diversity seems to be underestimated. Indeed, the interviewees were found very discreet when it was question of giving information on the indigenous medicinal knowledge of fungi. This discretion was also intensified when it was questioned to give information on how these medicinal fungi where used (ingredients, preparation, and posology). The mythical and religious purposes were not frequently reported by the interviewees. This result is in contradiction with those of some authors [40], who found a spiritual interaction between wild fungi and people of Tshopo Province in the Democratic Republic of the Congo.

A set of useful mushrooms were known and identified by all the visited sociolinguistical groups. Specific vernacular names were also attributed to all the encountered useful fungal species in every sociolinguistical group. These local names were usually found descriptive and giving information on the biotope, ecology, and substrate on which the fruit body occurs, the color, side of the fruit bodies, the taste, or the effect after consumption. This vernacular nomenclature was found similar to that of the Bofi pygmies in Central African Republic [5] and the Nagot of Central Benin [41]. This nomenclature differs from the conventional Latin one on the one hand and from one African region to another on the other hand [42]. Some confusions were also observed in the identifications of a set of sociolinguistical groups with species belonging to different genera but identified as a single species (i.e., presence of volva in the species of the genera *Amanita* and *Volvariella*). Furthermore, different species of the same genus were seen with a single vernacular name (e.g., *Termitomyces* species). These species were systematically called “Termite cultivated’s fungi” whatever the species. The most important criteria used for this vernacular nomenclature is the fructification of the species on a termite mound and the presence of a pseudorhiza in the mushrooms [7]. In short, a lack of structured and reliable vernacular nomenclature was observed during this work. This observation might also be due to (i) the lack of interest of current generations in mycological indigenous knowledge and (ii) the loss of fungal diversity caused by the permanent and continuous destruction of their habitats.

## Conclusion

The diversity of wild useful mushrooms varies from one phytogeographical zone to another and between sociolinguistical groups in Côte d’Ivoire. The use of fungal species also varied not only from one ethnic group to another but also from one visited village to another. It also appears that these useful mushrooms are known and used by rural people mainly as food and/or medicine. Species of the genus *Auricularia* were reported as the most useful mushrooms of the forest zone while *Termitomyces* spp. and *Psathyrella tuberculata* are the most used species of the Guinean and the Sudano-Guinean savanna zones. This country is one the fungi-rich West African country. However, the protected areas seem to be the last important sanctuaries of these organisms.

## Additional file

Additional file 1: **Table S1.** Vernacular nomenclature of the reported useful wild mushrooms in Côte d’Ivoire. (DOCX 41 kb)

## Abbreviations

CNP: Comoé National Park
CSI: Cultural Significance Index values
EUVk: Ethnomycological use value of a species for a category of use
MNP: Marahoué National Park
RUV: Reported use value
TNP: Taï National Park
