# Supplementary material for: Phytogeographical and sociolinguistical patterns of the diversity, distribution, and uses of wild mushrooms in Côte d’Ivoire, West Africa
Source: J Ethnobiol Ethnomed. 2019 Jan 18;15:5. doi: 10.1186/s13002-019-0284-5 (PMC6339280; doi:10.1186/s13002-019-0284-5)
Supplement: Supplementary file 1 — Table S1. Vernacular nomenclature of the reported useful wild mushrooms in Côte d’Ivoire. (DOCX 41 kb) [file 13002_2019_284_MOESM1_ESM.docx]

**Table S1:** Vernacular nomenclature of some of the reported useful wild mushrooms in Côte d’Ivoire. Meaning of some vernacular names are given in parentheses.

|  |  |  |  |  |  |  |  |  |
| --- | --- | --- | --- | --- | --- | --- | --- | --- |
| **Reported wild useful mushrooms** | **Families** | **Socio linguistic groups** | | | | | | |
|  |  | **Baoulé** | **Gouro** | **Guéré** | **Koulango** | **Lobi** | **Malinké** | **Oubi** |
| *Agaricus* sp1 | Agaricaceae | - | - | - | - | - | - | - |
| *Agaricus* sp2 |  | - | - | Café kanhou (Coffee plant’s fungus) | - | - | - | Gbôwoulo |
| *Amanita congolensis* (Beeli) Tulloss, B. E. Wolfe, K. W. Hughes, Kudzma & Arora | Amanitaceae | - | - | - | - | - | - | - |
| *Amanita masasiensis* Härk.& Saarim. |  | - | - | - | - | - | - | - |
| *Amanita strobilaceovolvata* Belli |  | - | - | - | - | - | - | - |
| *Amanita aff subviscosa* Belli |  | - | - |  | Sapouho | Gbancommine | Missi (Bull) | - |
| *Amanita craseoderma* Bas |  | - | - | - | - | - | - | - |
| *Amanita crassiconus* Bas |  | - | - | - | - | - | - | - |
| *Amanita subviscosa* Belli |  | - | - | - | Nadjein | Gbancommine | Massa fienan (God’s fungus) | - |
| *Amanita xanthogala* Bas |  | - | - | - | Nadjein | Noufécommine (King’s fungus) | Wôfienan | - |
| *Auricalaria* sp1 | Auriculariaceae | Gouro n’dré (Fungus of Gouro people) | Liblatenin (Old lady’s ear) | - | Kotingo (Devil’s ear) or Matingo (Dog’s ear) | Nougbêlêcommine | Wourou toro (Dog’s ear) | Urodohou (Monkey’s ear) |
| *Auricularia* *polytricha* ([Bull.](https://fr.wikipedia.org/wiki/Pierre_Bulliard)) [Quél.](https://fr.wikipedia.org/wiki/Lucien_Qu%C3%A9let) |  |  |  | Urodohou (Monkey’s ear) |  |  |  |  |
| *Auricularia* sp2 |  |  |  |  |  |  |  |  |
| *Auricularia* sp3 |  |  |  | Yourodohou (Monkey’s ear) |  |  |  |  |
| *Boletus loosii* Heinem. | Boletaceae |  |  |  | Savanyô | Gbancommine | Bâha (Goat) |  |
| *Bulgaria* sp. | Bulgariacea | Gnamiengbili n’zué sê (Water pot of God) | - | - | - | - | - | - |
| *Cantharellus addaiensis* Henn. | Cantharellaceae | - | - | - | Savanyô or Sirabasan | Natelcommine | Sirabasan |  |
| Cookeina sp1 | Sarcosyphaceae | - | - | Téhé | - | - | - | Urodohou (Monkey’s ear) |
| Cookeina sp2 |  | - | - |  | - | - | - |  |
| Coprinus africanus (Pegler) Redhead, Vilgalys & Moncalvo | Agaricaceae | N’gonisan | Ziberi or troutinin (Black fungus) | *Djokanhou* | Tanko Poubidigo (Black fungus) | Commine bissi (Black fungus) | Fiénanfi | Nénannon |
| *Daldinia* *concentrica* (Bolton) Cesati & de Notaris | xylariaceae | Waka n’dré (Wood fungus) | Yirimanoolou (Wood fungus) | Zué | Sousôron or Youko or yarayaradomolou | Yébriti or masouma (Navel’s medicine) | Baragro fiénan  (Navel’s fungus) | Nawibê |
| *Echinochaete brachypora* (Mont.) Ryvarden | Polyporaceae | Gbagbôtankan or Nananssougbô or Okessougbô | Sirabasan | Gbowoulo | Nantansi | Natelcommine | Sirabasan ou sirakôrôfienan | - |
| *Ganoderma lucidum* ([Curtis](https://fr.wikipedia.org/wiki/William_Curtis) ex [Fr.](https://fr.wikipedia.org/wiki/Elias_Magnus_Fries)) [P.Karst.](https://fr.wikipedia.org/wiki/Petter_Adolf_Karsten) | Ganodermataceae | - | - | - | Glalgbegougo (Monkey’s stool) | Térécommine | Soulawagané (Monkey’s stool) | - |
| *Ganoderma* sp. |  | - | - | Gblodohou (Elephant’s ear) | - | - | - | - |
| *Gyroporus castaneus* ([Bull.](https://fr.wikipedia.org/wiki/Pierre_Bulliard)) [Quél.](https://fr.wikipedia.org/wiki/Lucien_Qu%C3%A9let) | Gyroporaceae | - | - | - | Sabgô Nan | Loucommine or Gbancommine (Forest fungus) | Missi (Bull) | - |
| *Lactarius saponaceus* Verbeken | Russulaceae | - | - | - | Samme | Gbancommine | Missi (Bull) | - |
| *Lactarius tenellus* Verbeken &Walleyn |  | - | - | - | Sawôgô |  | Bâha (Goat) | - |
| *Lactifluus flammans* (Verbeken) Verbeken |  | - | - | - | Sa Nan | Loucommine (Forest’s fungus) | Fiékolo | - |
| *Lactifluus gymnocarpoides* (Verbeken) Verbeken |  | - | - | - | Sa nan Yelia | Gbancommine | Missi (Bull) | - |
| *Lactifluus heimii* (Verbeken) Verbeken |  | - | - | - | Sa nan Yelia |  | Yélia | - |
| *Lactifluus luteopus* Verbeken |  | - | - | - | Samme |  | Wôfiénén | - |
| *Lactifluus volemoides* (Karhula) Verbeken |  | - | - | - | Samme Tégué |  | Bâha (Goat) | - |
| *Lentinus squarrossulus* Mont. | Polyporaceae | Gouro n’dré (fungus of Gouro people) or okessougbô (Old lady’s ear) | Zahabi | Suhésuhé | Nantansi sirabasan | Natel commine | Sirabasan | - |
| *lentinus tuber-regium* (Fr.) Singer |  | Okessougbô (Grand parent’s ear) | Fouakannibô ou zahabi | Bglihekanhou | - | - | - | Gbôwoulo woi |
| *Lycoperdon* sp1 | Agaricaceae | Azihousoufoué or Ahoucotoiha | - | Pohopoho | - | - | - | - |
| *Lycoperdon* sp2 |  | Azihousoufoué or Ahoucotoiha | - | Pohopoho | - | - | - | - |
| *Marrasmiellus inoderma* (Berk.) Singer | Marasmiaceae | Wonzien n’dré (Flies’s fungus) or angouan n’dré | - | Péhé | - | - | - | Gnicloa |
| *Marasmiellus* sp. |  | - | Tiêbinnintrou or Boritrou (Twig fungus) | - | - | - | - | - |
| *Octaviana ivoryana* Casttelano, Verbeken & Thoen | Boletaceae | - | - | - | Sirabôfô | Gbricommine | - | - |
| *Psathyrella tuberculata* (Path.) A. H. Smith | Psathyrellaceae | N’dre blé (Black fungus) | Troutinin (Black fungus) | Gbaho | Poubidigo (Black fungus) | Commine bissi (Black fungus) | Fiénanfi  (Black fungus) | Woi co (Black fungus) |
| *Psathyrella* sp1 |  | N’gbégbé ou saki adja (Wedding cake) | Wawa | Gbaho | Poubidigo (Black fungus) | - | Fiénanfi or bougôlêlê (Black fungus) | Woi co ‘(Black fungus) |
| *Russula aff cellulata* Buyck | Russulaceae | - | - | - | Samme Yelia | Gbancommine | Bâhâ | - |
| *Russula cellulata* Buyck |  | - | - | - | Sahodjo | Gbancommine |  | - |
| *Russula ciliata* Buyck |  | - | - | - | Samme wouroh | Gbancommine |  | - |
| *Russula congoana* Pat. |  | - | - | - | Nanbanlourgo | - | Bawilé or linguêkrôfienan | - |
| *Russula grisea* Fr*.* |  | - | - | - | Yelia | Panté commine (Soil’s fungus) | Yelia | - |
| *Russula oleifera* Buyck |  | - | - | - | Yelia | Gbancommine | Yelia | - |
| *Russula sesenagula* Beeli |  | - | - | - | Yelia | Gbricommine | Linguêkrôfienan | - |
| *Russula* sp. |  | Dihiti | - | - | - | - | - | - |
| *Schizophyllum commune* Fries | Schizophyllaceae | - | - | Dokanhou (Rafia fungus) | - | - | - | - |
| *Termitomyces cf Aurantiacus* (R. Heim) R. Heim | Lyophyllaceae | - | - | *-* | Tafôtiho | Negban commine (Long stipe’s fungus) | Bagabadadjéhé  (Saliva of termite) or tafôti | - |
| *Termitomyces cf striatus* (Beeli) R. Heim |  | - | - | - | Tafôtiho | Negban commine (Long stipe’s fungus) | Bagabadadjéhé  (Saliva of termite) or tafôti | - |
| *Termitomyces* fuliginosus Heim |  | - | - | - | Gbaragbrô | Negban commine (Long stipe’s fungus) | Bagabadadjéhé  (Saliva of termite) or tafôti | - |
| *Termitomyces letestui* (Pat.) R. Heim |  | N’glo (With clay form) | Polonlon | - | - | - | - | - |
| *Termitomyces meduis* R. Heim & Grassé |  | Nandroh | Pbelehanin | Glahein | Tafotiho ou  soquant | Negban commine (Long stipe’s fungus) | Bagabadadjéhé  (Saliva of termite) or tafôti | Glalê |
| *Termitomyces microcarpus* (Berk. & Broomo) R. Heim |  | Wonzien n’dré (Flies’ fungus) | - | Glahein ou zacloa | - | - | - | Gli ou glalein |
| *Termitomyces schimperi* (Pat.) R. Heim |  | Groukagrou | - | Blahokanhou (Termites’ fungus) | - | - | - | Gbôwoulo |
| *Termitomyces* sp1 |  | Nandroh | Pbelehanin | Glahein | - | - | - | - |
| *Termitomyces* sp2 |  |  |  | - | - | - | - | - |
| *Termitomyces* sp3 |  |  |  | - | - | - | - | - |
| *Termitomyces* sp4 |  |  |  | - | - | - | - | - |
| *Termitomyces* sp5 |  |  |  | Glahein | - | - | - | - |
| *Termitomyces* sp6 |  |  |  | - | - | - | - | - |
| *Termitomyces* sp7 |  |  | - | - | - | - | - | Glalê |
| *Termitomyces* cf *clypeatus* |  |  | - | - | - | - | - | Glalê |
| *Termitomyces striatus* |  |  | - | - | - | - | - | - |
| *Volvariella earlei* (Murrill) Shaffer | Volvariellaceae | N’tê n’dré or havié n’dré (Riz’s bran fungus) or bohéfê akoua (False fungus) | Sabêtrou (Riz’s bran fungus) | Cofrikanhou (Riz’s bran fungus) | - | - | - | Touhotou woi (Rice’s bran fungus) |
| *Volvariella volvaceae* (Bull.) Singer |  | - | Tienbowa (Gold fungus) | Gbola ou gbohokanhou (Palm tree fungus) | Tienkopouho (Palm tree fungus) | - | - | Djowoi (Palm tree fungus) |
| *Volvariella* sp. |  | Bôhefê akoua (Sweet false fungus) | Boha | - | - | - | Kôhi | - |
